# Injuries in Collegiate Women’s Volleyball: A Four-Year Retrospective Analysis

**DOI:** 10.3390/sports5020026

**Published:** 2017-05-10

**Authors:** Christopher J. Sole, Ashley A. Kavanaugh, Michael H. Stone

**Affiliations:** 1 Department of Health, Exercise, and Sport Science, The Citadel, The Military College of South Carolina, Charleston, SC 29409, USA; 2 Center of Excellence for Sport Science and Coach Education, Department of Sport, Exercise, Recreation, and Kinesiology, East Tennessee State University, Johnson City, TN 37614, USA; kavanaugh.ashley@gmail.com (A.A.K.); stonem@mail.etsu.edu (M.H.S.)

**Keywords:** injury, time-series, athlete monitoring, volleyball

## Abstract

A four-year retrospective analysis of injury data was conducted on a collegiate (NCAA Division I) women’s volleyball team. Twenty athletes (Year 1: age = 19.4 ± 0.9 y, height = 175.2 ± 5.1 cm, body mass = 70.5 ± 10.2 kg; Year 2: age = 20.1 ± 1.0 y, height = 175.7 ± 4.7 cm, body mass = 69.5 ± 10.1 kg; Year 3: age = 20.1 ± 1.4 y, height = 173.8 ± 6.3 cm, body mass = 69.9 ± 10.8 kg; Year 4: age = 19.5 ± 1.4 y, height = 174.4 ± 8.6 cm, body mass = 72.7 ± 10.8 kg) participated in this study, accounting for 1483 total training exposures. Injury was defined as any damage to a body part, incurred during volleyball or strength and conditioning-related activities, which interfered with training and/or competition. Injury rate was normalized to the number of athletes and exposure and expressed as injuries per 1000 exposures. A total of 133 injuries were recorded. The most common injury was to the knee (left = 7.5%, right = 12.0%). Injuries occurred most often in volleyball practice (75.2%), followed by competition (20.3%), and strength and conditioning-related activities (4.5%). Non-contact injuries (upper body = 26.3%, lower body = 53.4%) were more common than contact injuries (upper-body = 13.5%, lower-body = 6.8%). An examination of injury rates relative to the training year revealed patterns in injury occurrence. Specifically, spikes in injury rate were consistently observed during periods of increased training volume that were preceded by breaks in organized training, such as the early pre-season and off-season training periods.

## 1. Introduction

Whether mild or severe, injuries can be detrimental to the training process and an athlete’s career, resulting in lost playing time [1,2,3], and potentially negate an athlete’s or team’s competitive success. Injury research in sports is useful for providing coaches, sport medicine, and strength and conditioning professionals with information pertaining to the common mechanisms and frequency of injury. Perhaps the most valuable purpose of injury research is to provide insight that may lead to injury prevention. 

Injuries in volleyball are most commonly attributed to jumping, landing, hitting, and blocking movements, with the majority of acute and overuse injuries occurring from jumping [4]. Additionally, more injuries occur during hitting and blocking tasks than passing and setting in volleyball [4]. Epidemiological research on volleyball-related injuries has been performed both retrospectively [5,6,7,8,9] and prospectively [10,11,12]. Retrospective research has primarily focused on determining mechanisms, locations, and frequencies of incurred injuries, whereas prospective research has focused on examining injury prevention strategies and risk of re-injury. In general, similar injury patterns have been reported between male and female volleyball athletes [10,12]. However, it should be noted that sex has been identified as a risk factor for some common volleyball injuries [13] such as patellar tendinopathy “jumper’s knee” in males [14,15] and glenohumeral subluxation in females [16].

Despite its popularity, few injury studies have specifically examined women’s collegiate volleyball [7,17]. Participation at the collegiate level is influenced by unique constraints imposed by the NCAA, such as the regulation of organized training throughout the academic year. Considering these constraints, additional research specifically involving collegiate athletes is warranted. Researchers should seek to further understand the distribution of injuries, the activities where injuries take place, and identification of specific time periods where these athletes may be more susceptible to injuries. Therefore, the purpose of this study was to retrospectively examine the frequency, distribution relative to activity, and relative timing of injuries in a National Collegiate Athletic Association (NCAA) Division I women’s volleyball team over four academic years.

## 2. Materials and Methods

A four-year retrospective analysis of injury data from a collegiate women’s volleyball team was performed. All data were collected as part of an ongoing athlete performance monitoring program [18]. The analysis examined data from four consecutive academic years. Each academic year began in August and ended the following May. Athletes were not monitored during the months of June and July while away from university on summer break. This academic year was chosen over the calendar year because first-year and/or transfer athletes joined the team each August at the beginning of the team’s pre-season—a few weeks before the team’s first regular season competition. The team’s head coach and the Universitiy’s Director of Sports Medicine remained the same for the duration of the study.

### 2.1. Participants

Data from twenty collegiate (NCAA Division I) women’s volleyball athletes were included in this analysis (Year 1: age = 19.4 ± 0.9 y, height = 175.2 ± 5.1 cm, body mass = 70.5 ± 10.2 kg; Year 2: age = 20.1 ± 1.0 y, height = 175.7 ± 4.7 cm, body mass = 69.5 ± 10.1 kg; Year 3: age = 20.1 ± 1.4 y, height = 173.8 ± 6.3 cm, body mass = 69.9 ± 10.8 kg; Year 4: age = 19.5 ± 1.4 y, height = 174.4 ± 8.6 cm, body mass = 72.7 ± 10.8 kg). All athletes had previous experience competing in volleyball prior to their collegiate careers. The study was conducted retrospectively using archived data, and the scope and methodology of this study was reviewed and approved by the Research Ethics Committee of East Tennessee State University.

### 2.2. Data Analysis

The present study defined injury as “any damage to a body part incurred during volleyball, resistance training, or other conditioning-related activity, which interfered with training and/or competition.” This criterion was selected based on previous research [19,20], and attempts to represent all severities of injuries. For example, it is not uncommon for a volleyball athlete to sustain a minor injury during training or competition that allows them to continue participation, but with limitations (modifications to exercise type, range of motion, volume and intensity of exercise). A more narrow definition of injury such as one based on time-loss (i.e., missed training days) only, might underrepresent many less severe injuries that still allow for participation, albeit at a reduced capacity. Minor injuries that do not result in missed training days may still have a profound impact on an athlete’s long-term development due to a reduction in intensity and overall training volume. Therefore, this investigation used a broad definition of injury to better gauge its effect on long-term athlete development. 

Hours of strength and conditioning-related activities, volleyball practice, and competition were obtained from records kept by the coaching staff and athletic administration. All injuries were evaluated, recorded, and classified by a certified athletic trainer at the time of occurrence. Injuries were recorded using a web-based data management system (SportsWareOnLine™, Computer Sports Medicine Inc., Stoughton, MA, USA). Following retrieval from the database, all injury data were de-identified prior to any analysis. Injury rates were calculated by dividing the number of injuries by the total number of exposures (each exposure was equal to one hour of strength and conditioning-related activities, volleyball practice, or competition). The time of each exposure was recorded to the nearest fifteen minutes. Injury rate was normalized to the number of athletes and exposures, and was expressed as injuries per 1000 exposures. Therefore, the incidents of injuries were expressed relative to total exposures, including all athletes and all competition and training-related activities. 

Injuries were further classified as “acute” or “overuse” and “contact” or “non-contact”. This investigation defined acute as any injury resulting from a single, identifiable, trauma or event [21]. Injuries resulting from repeated microtrama, absent of a single, identifiable event were classified as overuse [21,22]. Injuries suffered from contact occurred as a result of colliding with a teammate, opposing player, or an object. Non-contact injuries were any injuries occurring in the absence of contact with another player or object. All injury classifications were made by sports medicine personnel at the time of occurrence and/or during the subsequent days following injury. 

**Figure 1:**
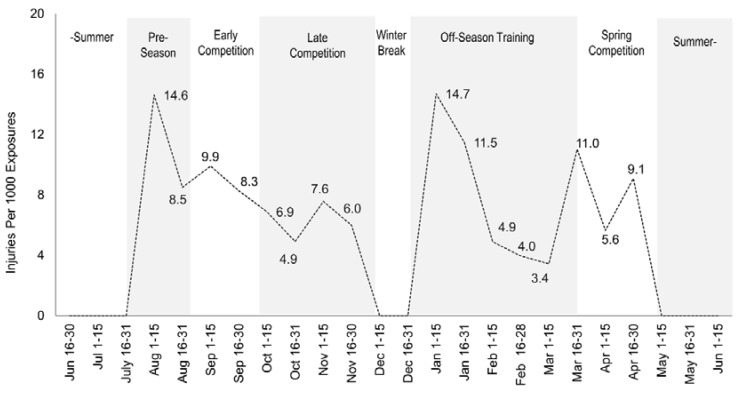
A descriptive time-series plot displaying injury rates over four academic years. Data are collapsed into a single academic year and displayed relative to common phases of training and competition found in NCAA Division I women’s volleyball. Note: data are displayed at approximately a 15-day resolution.

A descriptive time-series plot was constructed to examine common injury patterns as well as to gain insight into the specific times throughout the academic year injury rates were the highest. Each year was divided into twenty-four (approximately 15-day) periods. Injury rate data from each period of each year were combined and then collapsed into a single year. The single year was divided into seven periods based on the common phases of training and competition found in collegiate women’s volleyball. Injury rate data were plotted with the *x* axis representing time to visually examine any existing patterns (Figure 1). All data analyses were performed using Microsoft Excel 2013 (Microsoft Corporation, Redmond, WA, USA).

## 3. Results

**Figure 2:**
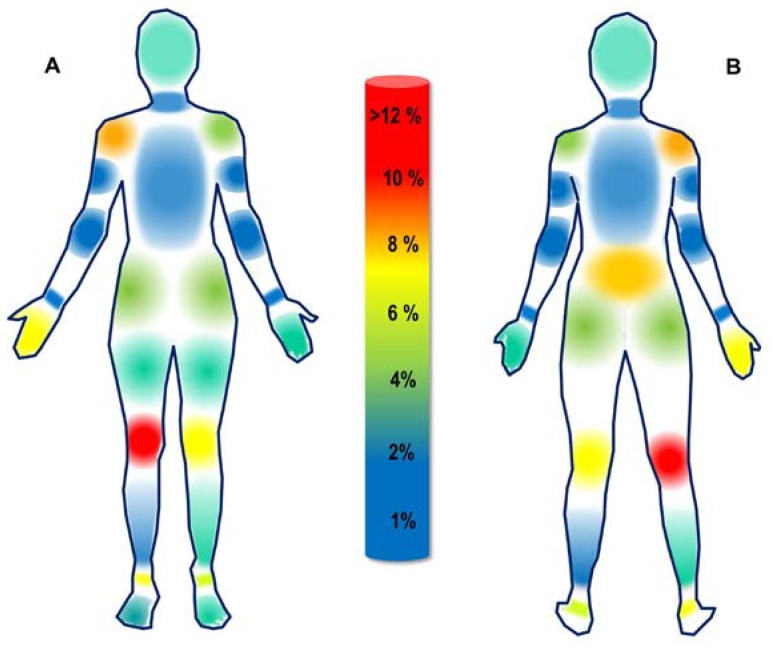
Heat map displaying the relative distribution of injuries per body region over four academic years: (**A**) Anterior view; (**B**) Posterior view.

A total of 1483 exposures were accumulated by twenty athletes over a four-year period. From the total exposures, 133 injuries were incurred from competition and training-related activities. Annual injury rates ranged from 5.1 (Year 1) to 11.6 (Year 2) injuries per 1000 exposures. Table 1 and Figure 2 display the frequency and distribution of total injuries per body region over a four-year period. Table 2 displays the frequency, total injury rate, and distribution of injuries by activity. Table 3 displays the frequency and distribution of injuries by type and source. 

Visual inspection of the time-series plot revealed that the highest injury rates appeared to occur during the pre-season (early August) and off-season training (early January) (Figure 1). Overall, injuries decreased throughout the competitive season (late August to late November) and off-season (late January to early March) after their initial peaks. Injury rates also showed an abrupt increase during the spring competitive season (late March to late April), although not as great as the fall (primary) competitive season.

## 4. Discussion

The purpose of this retrospective analysis was to identify the type, frequency, and relative timing of injuries within a NCAA Division I women’s volleyball team. The primary findings of this analysis are (1) specific body regions such as the knee and shoulder accounted for a large percentage of total injuries observed; (2) the majority of the injuries were classified as non-contact and occurred most often in a volleyball practice setting; and (3) the highest injury rates consistently occurred following breaks from organized training (i.e., summer and winter recess).

When discussing injury rates, comparison between studies can be difficult and must be approached with caution as methodology between studies may vary considerably. The present study reported 8.40 injuries per 1000 exposures over a four-year period. Data from the NCAA injury surveillance system (ISS) report an overall injury rate in women’s volleyball of 4.3 (2004–2005 to 2008–2009 academic years) [23] and 4.06 (2005–2006 to 2008–2009 academic years) [17] injuries per 1000 exposures. The elevated injury rate observed in the present analysis could potentially be the result of several factors including inconsistencies in study methodology. For example, prior to the 2009–2010 academic year, the NCAA ISS only considered injuries that resulted in the loss of one or more days of sport participation [24]. This analysis used an intentionally broad operational definition of injury. It is possible that existing reports may not have included many minor injuries that were factored into this analysis. For this reason, standardization of injury definitions as well as data collection procedures in injury research has been stressed [21,25,26]. Additionally, authors have suggested classifying injury severity by functional level and not time-loss to better represent all types of injuries [22].

In this study, the most frequently injured body regions were the knee, ankle, and shoulder, consistent with previously published literature on men’s and women’s volleyball athletes [6,10,12,27,28]. It is likely that these injuries are related to the repetitive jumping, high-force landing, and overhead striking movements, characteristic of indoor volleyball [4,29,30]. The majority of total injuries were classified as acute (69.9%) non-contact (79.7%) and occurred during volleyball practice (75.2%). Contact injuries generally occurred as a result of an athlete colliding with an opposing player, teammate, or the ball. A common region for contact injuries was the hand/finger region. The high injury frequency of this region has been associated with blocking and hitting movements used in volleyball [31,32], and may be a consequence of trauma that is developed throughout one or many practice and training sessions. Additionally, this analysis found a disproportionate amount of injuries occurring in athlete’s right shoulders and right hand/finger region. Although beyond the scope of the present analysis, it is probable that this finding is simply the result of having a greater proportion of right-hand dominate athletes (i.e., right-handed hitters). Roughly 30% of the injuries observed were classified as overuse, or not attributed to a single identifiable event. The remaining injuries were classified as acute and associated with a single, identifiable trauma. Typically, overuse injuries occur at a lower rate in volleyball as compared to acute injuries [33]. However, accurately quantifying overuse injuries is challenging [22]. Thus, the prevalence of overuse injuries may be underestimated [34]. Regardless, understanding overuse injury in volleyball is important, as many risk factors for sustaining these injuries may be modifiable [13,35].

A novel aspect of this study was the high-resolution examination of the relative timing of injury over the four-year period (Figure 1). When injury rates were plotted relative to the training year, interesting patterns were identified. Injury literature on collegiate women’s gymnastics has demonstrated that the highest injury rates occur later in the competitive season, likely due to the accumulation of fatigue [20]. One might assume that injury rates in volleyball would exhibit a similar behavior. However, according to the visual analysis of the time-series plot, this study demonstrates that the highest injury rates occur during the early pre-season (August) and early off-season (January). These finding are in accordance with an overview of injury statistics reported by the NCAA stating that the pre-season has the highest overall injury rate (6.5 per 1000 exposures), as compared to the in-season injury rate of 3.6 injuries per 1000 exposures in collegiate women’s volleyball [23]. Interestingly, the same report found that the post-season had the lowest rate of injuries (2.4 per 1000 exposures). This study found that the lowest rate of injuries throughout the entire year occurred in the off-season with the exception of a noticeable spike in the early off-season. Interestingly, each of the observed spikes in injury occurrence were preceded by periods of unmonitored or little training when the athletes were away from university (i.e., summer and winter breaks). Additionally, the training periods where these spikes occur are typically characterized by abrupt increases in training volume and intensity. Although this study lacks direct evidence to establish causational relationships, it is possible that detraining as a result of periods of inactivity or decreased activity during winter break, contributed to the spike in injury rates [36,37]. A similar scenario is possible following summer break prior to the pre-season in early August. Athletes who were noncompliant to the training program may be especially vulnerable to injuries during this time of year due to the high practice workloads undertaken. In NCAA Division I women’s volleyball, a pre-season training period is approximately three weeks, a relatively short amount of time to prepare physically, technically, and tactically for the upcoming competitive season [38]. During this pre-season period, it is not uncommon for the athletes to practice up to three times per day. Decreased fitness, combined with high practice workloads may be responsible for an increased injury rate during this time [39]. A second factor for the abrupt increase in injury rate early in each academic semester could be attributed to elevated outside stressors; a factor that some researchers have attempted to link to incidence of injuries [40,41,42,43,44]. Outside stressors such as leaving home, beginning new classes, and joining a new team, may contribute to additional stress, resulting in an increased vulnerability to injuries.

Although the results of this study may provide unique insight into the relative timing of injury as well as corroborate the results of previous investigations, the present study includes several limitations. First, the results of the study are based on observations from a single team. Therefore, caution should be exercised in attempting to generalize this information. Additionally, the composition of the team changed over the four-year period as players joined and left the team. Finally, the injury data contained in this analysis were delimited to location and mechanism of injury. Thus, this study provides only a general overview of injury location and timing. Future research should consider examining the relative timing of specific injuries in collegiate women’s volleyball. Additionally, future research should examine relationships between workload, fitness, and injury, as this information may provide the greatest insight related to injury prevention/minimization.

## 5. Conclusions

In conclusion, the present study reports findings that are congruent with the existing literature on NCAA collegiate women’s volleyball athletes and injuries [7,17,22]. Collegiate women’s volleyball athletes are particularly susceptible to acute non-contact injuries to the knee, shoulder, ankle and lower back. A novel finding of this study relates to the relative timing of injury throughout the training year. The data presented in this study would suggest that these athletes are especially susceptible to injury during early pre-season and the off-season after returning from summer and winter breaks spent away from university. Consequently, coaches and practitioners should be mindful to manage intra- and inter-session fatigue, and use fatigue management strategies in an attempt to decrease the frequency of acute non-contact injuries incurred during volleyball practice and other training activities. Similarly, volleyball coaches, strength and conditioning staff, and sport medicine personnel should work together to establish proper training progressions (frequency, volume, and intensity) of all training parameters, starting with lower workloads when the athletes return from breaks, and gradually progressing to higher workloads once the athletes are adequately prepared. Moreover, commitment to following a periodized year-round strength and conditioning program should be emphasized to the athletes in order to minimize injuries and improve volleyball-related fitness characteristics. 

## Figures and Tables

**Table 1:** Frequency and percentage of total injuries incurred over four academic years.

	**Frequency**	**% of Total**		
**Head**	3.0	2.3		
**Neck**	2.0	1.5		
**Torso**	3.0	2.3		
**Lower Back**	10.0	7.5		
**Area Total**	18.0	13.6		
	**Left Side**		**Right Side**	
	**Frequency**	**% of Total**	**Frequency**	**% of Total**
**Shoulder**	5.0	3.8	11.0	8.3
**Upper Arm**	1.0	0.8	0.0	0.0
**Elbow**	1.0	0.8	1.0	0.8
**Wrist**	2.0	1.5	2.0	1.5
**Hand/Finger**	4.0	3.0	9.0	6.8
**Hip**	5.0	3.5	6.0	4.3
**Thigh**	3.0	2.3	4.0	3.0
**Knee**	10.0	7.5	16.0	12.0
**Shin**	4.0	3.0	1.0	0.8
**Calf**	1.0	0.8	3.0	2.3
**Ankle**	7.0	5.3	10.0	7.5
**Foot**	4.0	3.0	5.0	3.8
**Area Total**	47.0	35.3	68.0	51.1
**Grand Total**	133.0	100.0		

**Table 2:** Frequency and percentage of total injuries based on activity.

Activity	Frequency	% of Total	Injuries per 1000 Exposures
**Practice**	100.0	75.2	11.6
**Competition**	27.0	20.3	6.6
**Strength and Conditioning**	6.0	4.5	1.7
**Total**	133.0	100.0	

Note: An exposure equals 1 h of strength and conditioning related activities, volleyball practice or competition.

**Table 3:** Frequency and percentage of total injuries incurred based on type.

Type	Frequency	% of Total
**Non-Contact Upper-Body**	35.0	26.3
**Non-Contact Lower-Body**	71.0	53.4
**Contact Upper-Body**	18.0	13.5
**Contact Lower-Body**	9.0	6.8
**Acute**	93.0	69.9
**Overuse**	40.0	30.1
